# The Role of Deformation and Microstructure Evolution on Texture Formation of a TA15 Alloy Subjected to Plane Strain Compression

**DOI:** 10.3390/ma17153752

**Published:** 2024-07-29

**Authors:** Xianxian Wang, Xin Jia, Wenhao Wu, Jun Cheng, Xueni Zhao, Mingjie Shen

**Affiliations:** 1 School of Mechanical and Electrical Engineering, Shaanxi University of Science and Technology, Xi’an 710021, China; 18220208425@163.com (X.J.); 220511033@sust.edu.cn (W.W.); zhaoxueni@sust.edu.cn (X.Z.); smjiekaka@163.com (M.S.); 2 State Key Laboratory of Materials Processing and Die & Mould Technology, Huazhong University of Science and Technology, Wuhan 430074, China; 3 Shaanxi Key Laboratory of Biomedical Metal Materials, Northwest Institute for Nonferrous Metal Research, Xi’an 710016, China

**Keywords:** TA15 titanium alloy, plane strain compression, texture evolution, microstructure evolution, microhardness

## Abstract

In this study, the texture formation mechanism of a TA15 titanium alloy under different plane strain compression conditions was investigated by analyzing the slipping, dynamic recrystallization (DRX) and phase transformation behaviors. The results indicated that the basal texture component basically appears under all conditions, since the dominant basal slip makes the C-axis of the α grain rotate to the normal direction (ND, i.e., compression direction), but it has a different degree of deflection. With an increase in deformation amount, temperature or strain rate, {0001} poles first approach the ND and then deviate from it. Such deviation is mainly caused by a change in slip behaviors and phase transformation. At a smaller deformation amount and higher strain rate, inhomogeneous deformation easily causes a basal slip preferentially arising from the grain with a soft orientation, resulting in a weak basal texture component. A greater deformation amount can increase the principal strain ratio, thereby promoting other slip systems to be activated, and a lower temperature can increase the critical shear stress of the basal slip, further causing a dispersive orientation under these conditions. At a higher temperature and a lower strain rate, apparent phase transformation will induce the occurrence of lamellar α whose orientation obeys the Burgers orientation of the β phase, thereby disturbing and weakening the deformation texture. As for DRX, continuous-type (CDRX) is most common under most conditions, whereas CDRX grains have a similar orientation to deformed grains, so DRX has little effect on overall texture. Moreover, the microhardness of samples is basically inversely proportional to the grain size, and it can be significantly improved as lamellar α occurs. In addition, deformed samples with a weaker texture present a higher microhardness due to the smaller Schmidt factors of the activated prism slip at ambient loading.

## 1. Introduction

**Figure 1:**
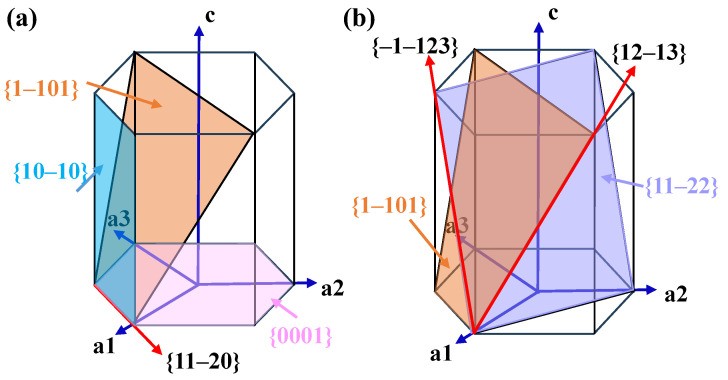
Slip systems of α-Ti: (**a**) <a> slip systems; (**b**) <a + c > slip systems.

With the rapid development of aerospace and ocean engineering and other high-end equipment, there is an urgent demand for their key components to have good hot workability, thermal strength and high temperature service performance, such as the aircraft’s overall spacer frame and the central wing under the wall plate. Near-α titanium alloys are widely used in such components because they combine the advantages of good hot workability, thermal strength and weldability of α and (α + β) titanium alloys, and possess high room-temperature and medium-temperature strength [1,2,3]. In addition, these components are always utilized as critical load-bearing structures and serve in extreme environments, so high performance in all directions must be possessed. However, for an α phase with a hexagonal close-packed (HCP) structure, there are limited slip systems, as shown in Figure 1. During thermal mechanical processing (TMP), preferential slip behavior easily results in a significant deformation texture, further leading to anisotropic mechanical properties of the components, which seriously restricts their service performance [4]. In addition, TMP involves many processing parameters, whose interaction effect can intensify the complexity of the evolution of texture and mechanical properties. Therefore, how to weaken the deformation texture and tailor the anisotropic mechanical property has become a frontier scientific problem for near-α titanium alloys during TMP.

Generally, the texture component strongly depends on the plastic deformation mechanism, i.e., slip and twin on the one hand [5,6,7,8], since the slip or twin activity is always accompanied by lattice rotation during deformation. Zhao et al. [8] analyzed the correlation between the activated slip systems and grain rotation behaviors of α-Ti by combining numerical simulation and experiment. They found that the basal slip can cause the α grain to rotate around the [10-10] axis; the prism slip can make the α grain rotate around the [0002] axis; and the pyramidal <a + c> slip will promote the α grain to rotate around the [10-10] axis. On the other hand, the texture component of titanium alloy is closely related to the microstructure evolution, i.e., recrystallization and phase transformation, because these phenomena can induce newly oriented grains during TMP [9,10,11,12,13,14,15,16,17]. For example, Wagner et al. [14,15] found that during the annealing heat treatment of cold-rolled titanium plates, only partial grains that produce deformation twins underwent an orientation change at the initial static recrystallization (SRX) stage. In addition, recrystallization texture is formed due to the oriented growth of the recrystallized grain, which is dependent on the combined effect of the deformed grain size, orientation and grain boundary features. Wang et al. [16] analyzed the effect of SRX on the texture of TA15 titanium spun parts under different pre-strains and annealing temperatures. The results indicate that discontinuous static recrystallization (DSRX) is prevalent at smaller pre-strains and DSRX grains with {11-20} axes parallel to the normal direction preferentially grow by long-range migration through 60~90° grain boundaries (GBs). With increasing pre-strain, the dominant mechanism gradually changes from DSRX to continuous static recrystallization (CSRX), showing an obvious non-standard basal texture. Ma et al. [17] studied the influencing factors to obtain a strong phase transformation texture of TA2 pure titanium. The results indicated that deformed β, recrystallized β and transformed β can develop a strong {11-20} texture, and deformation can obviously facilitate the generation of a {11-20} transformation texture. Based on these results, many investigations on the texture tailoring of titanium alloys have been carried out by optimizing the deformation paths or process conditions to change the plastic deformation mechanism or introducing recrystallization and phase transformation [18,19,20,21,22]. For example, Li et al. [18] found that when rolling a Ti60 alloy, a unidirectional rolling process at 980 °C would produce a T-type texture, while cross-rolling would cause a B-type texture. Han et al. [19] investigated the role of initial grain size on the recrystallization texture of rolled Mg-3Gd (wt.%) alloys. The results show that there are different preferential nucleations in the early stage of recrystallization, resulting in different texture characteristics. In addition, our team explored the influence of strain path on texture evolution. The results show that unidirectional torsion can produce an intense shear texture of a TA15 alloy, while forward/reverse torsion can obviously weaken the deformation texture owing to lattice recovery and the prevalence of continuous dynamic recrystallization. It can be found that the above studies on texture tailoring mainly focus on one aspect, i.e., deformation mechanism or microstructure evolution, while how the two factors together affect the texture is still unclear. Therefore, it is necessary to understand the influence of the deformation mechanism and microstructure evolution on texture formation under different forming conditions.

For this purpose, the texture and microstructural phenomena of a TA15 alloy under different plane strain compression conditions were investigated, and the influences of the deformation mechanism and microstructure evolution on texture formation were further discussed. Finally, the role of microstructure and texture in microhardness under various deformation conditions was analyzed. The results can provide a foundation for the texture controlling and mechanical property optimizing of titanium alloy components during TMP.

## 2. Experimental Procedures

**Figure 2:**
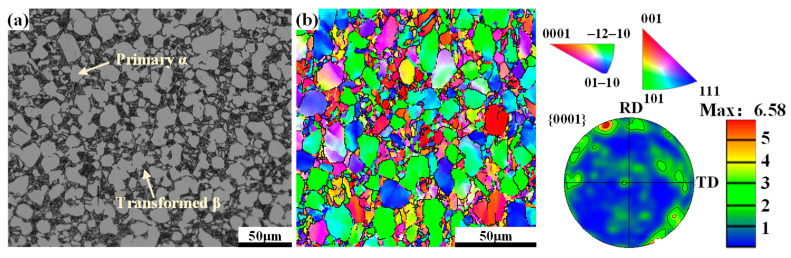
Microstructure and orientation features of the initial billet: (**a**) optical microstructure; (**b**) inverse pole figure (IPF) and {0001} poles of α grains.

In this work, a TA15 near-α titanium alloy is used, which is made of Western Superconducting Materials Technology Co., LTD. in China. The main composition of the alloy is reported in Reference [4]. The α/β phase transition temperature of the alloy is around 998 °C. The initial microstructure and orientation features of the billet are shown in Figure 2. It can be seen that there is an obvious equiaxed microstructure in the initial billet. Among the features, the volume fraction of coarse primary α (α_p_) grains is around 52.95%, and the mean size of the grain is around 12.86 μm. In addition, there is a weak cluster zone of {0001} poles which mainly converges in the normal direction (ND).

**Figure 3:**
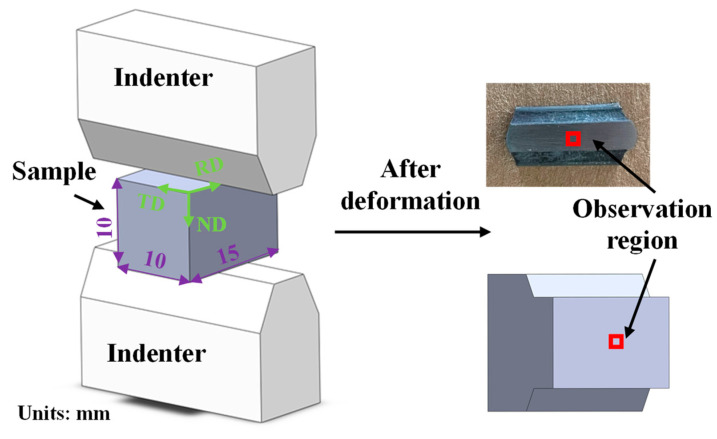
The schematic diagram of the plane strain specimen and characterized region.

The plane strain compression experiments were carried out on a Gleeble-3500 thermal simulation testing machine (DSI Company, St. Paul, MN, USA). The dimensions of the specimen and indenter distribution are displayed in Figure 3. Here, considering the strain state of plane strain compression is similar to that of the rolling process, the coordinate system in the plane strain compression was set to that of rolling (Figure 3). During deformation, the specimen was first heated to a temperature of 800 °C at a rate of 10 °C/s and kept there for 5 min, and then compressed. The detailed compression conditions are presented in Table 1. After deformation, the specimen was cut perpendicularly to the rolling direction (RD). Then, the microscope features in transverse direction (TD)–normal direction (ND) plane were characterized. The microstructural specimens were prepared by grinding and electrolytic polishing. The electrolytic polishing was performed at a voltage of 30 V and temperature of 5 °C for 45 s in a solution of 5% HClO_4_ + 65% CH_3_OH + 30% C_4_H_10_O. Subsequently, an FE-SEM Merlin (Zeiss Gemini 300, Carl Zeiss AG, Oberkochen, GER) compact scanning electron microscope from Zeiss was used for Electron Back-Scattered Diffraction (EBSD, (Oxford NordlysNano, High Wycombe, UK)) testing, and the data were analyzed using HKL Channel 5 software.

In addition, Vickers hardness tests were performed in the central region of the TD–ND plane of the sample through a THV-1MD (Chengdu Qifeng Technology Co., LTD., Chengdu, Sichuan, China) equipment. The applied load was 200 g and the pressure holding time was 10 s. In addition, Vickers hardness was tested 10 times under each forming condition and the average value was taken as the result.

## 3. Results and Discussion

### 3.1. Texture Evolution Mechanism under Different Deformation Conditions

#### 3.1.1. The Effect of Deformation Amount

**Figure 4:**
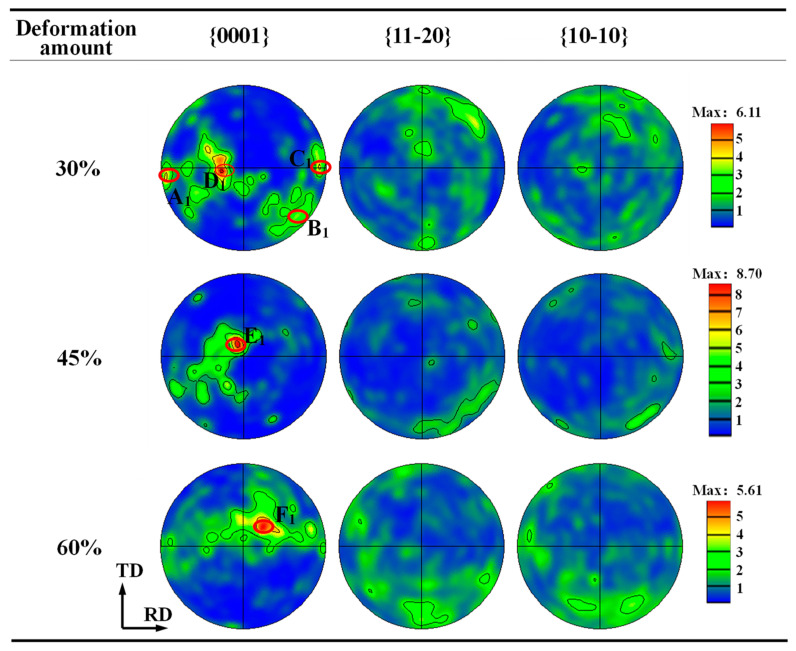
PF of α phase under different plane strain compression deformation amounts.

Figure 4 shows the pole figure (PF) of the α phase under different plane strain compression deformation amounts at a temperature of 850 °C and a strain rate of 0.01 s^−1^. It can be found that the {0001} poles of the α grains are mainly distributed near the ND, while the {10-10} and {11-20} poles are located on the RD-TD plane, exhibiting a distinct basal texture component regardless of deformation amount. When the deformation amount is 30%, the poles are distributed dispersively, and the {0001} poles are mainly clustered in zones A_1_, B_1_, C_1_ and D_1_, of which the intensity in zone D_1_ is the largest. When the deformation amount increases to 45%, the orientation concentration of the α grains is obviously enhanced, and most {0001} poles are located near the ND. The strongest cluster zone, E_1_, has an angle of 11° with the ND and an angle of around 20° with the TD. As the deformation amount is increased to 60%, most {0001} poles (F_1_) are still near the ND, while the deflection degree of {0001} from the ND in this case is increased compared to that under a small deformation amount.

The above orientation features first result from the slip behavior during deformation. For α titanium alloys, basal slip is the dominating deformation mechanism in the two-phase region [23,24,25,26,27,28]. Under a significant compressive deformation state, the activated basal slip can make the C-axis of the α phase gradually deflect towards to the compression axis [7]. Therefore, when the deformation amount is small, partial grains with preferential orientation are first rotated accompanied with basal slipping, thereby causing dispersive poles in this case. When the deformation increases, a large compressive strain along the ND leads to the basal texture forming. However, since the c/a ratio of α-Ti is 1.587, which is less than the 1.633 of the ideal HCP structure, the deformation texture will deviate from the ND slightly [5]. In addition, compared with ordinary unidirectional compression, the strain in the TD is limited under the present strain state, so the {0001} pole will be deflected, and the texture will not be the standard basal texture. Furthermore, the deflection angle between the C-axis and the ND depends on the activation ratio of each slip system and the principal strain ratio in each direction. Generally, in a plane strain compression state, a greater deformation amount causes a higher principal strain ratio, further resulting in a more significant deflection of the basal texture [8]; thus, the deflection degree under 60% deformation amount is obvious.

**Figure 5:**
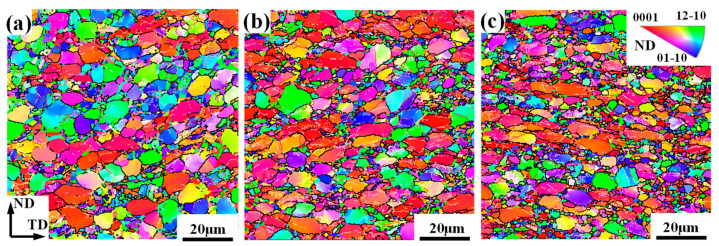
IPF maps of a TA15 alloy under different plane strain deformation amounts: (**a**) 30%; (**b**) 45%; and (**c**) 60%.

**Figure 6:**
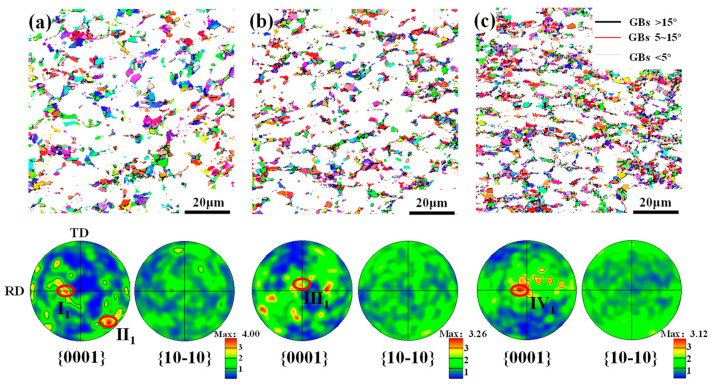
DRX grains and their {0001} and {10-10} PFs of a TA15 alloy under different plane strain deformation amounts: (**a**) 30%; (**b**) 45%; and (**c**) 60%.

In addition, to explore the effects of microstructural phenomena on the texture evolution, IPF maps under different deformation amounts were obtained, as shown in Figure 5. In these maps, high-angle grain boundaries (HAGBs, >15°), medium-angle grain boundaries (MAGBs, 5–15°) and low-angle grain boundaries (LAGBs, <5°) were marked as black, red and white line, respectively. It can be seen that with the increase in deformation, most α_p_ grains are gradually flattened along the ND, thus exhibiting a greater axial ratio. Moreover, a large number of refined grains are produced and their amount increases as the deformation amount increases. Such refinement was attributed to DRX [11], so the DRX grain was characterized through the grain orientation spread method, as shown in Figure 6. Here, the region less than 3° is considered as DRX grains. Through quantitative statistics, it is found that the kinetic of DRX increases with deformation and it can increase up to 38% at a 60% deformation amount. Correspondingly, the mean size and volume fraction of α_p_ is obviously reduced.

Figure 6a–c gives the orientation characteristics of DRX grains. It can be found that when the deformation amount is small, i.e., 30% and 45%, there are many clustered zones of {0001} poles, and zones I_1_, Ⅱ_1_ and Ⅲ_1_ are similar to those of overall grains. Meanwhile, the distribution of the {10-10} pole is rather scattered. As the deformation amount increases to 60%, although the orientation of DRX grains is dispersed, the clustered zone Ⅳ_1_ of the {0001} pole is similar to that of the deformed grains. For the TA15 titanium alloy, both discontinuous DRX (DDRX) and continuous DRX (CDRX) can be observed during thermal deformation. As we all know, for DDRX, new grains are formed through nucleation and growth at the expense of the region full of dislocation. While for CDRX, new grains are generated through the progressive transformation of LAGBs to HAGBs. Generally, the orientation of DDRX grains differs from that of deformed grains, whereas the orientation of CDRX grains is close to that of deformed grains. By combining the orientation of overall grains and the DRX grain shown in Figure 4 and Figure 6, and the microstructure features illustrated in Figure 5, it can be inferred that both DDRX and CDRX occur during the plane strain deformation of the TA15 alloy. However, with the increase in deformation, CDRX is gradually more common. The detailed reasons are as follows. Under a small deformation amount, slipping occurs first in the grain with a favorable orientation and it is impeded by GBs when the stored energy is rather high. So, inhomogeneous deformation is conducive to DDRX nuclei and growth. With an increase in deformation, a large number of slip systems are activated and plenty of substructures are generated within the grain. To coordinate plastic deformation, lattice rotation or misorientation accumulation of the substructure occurs, which promotes the prevalence of CDRX. However, the prevalence of CDRX grains still has little influence on the overall texture evolution since there is a similar orientation of the grain to the deformed grain, as shown in Figure 4 and Figure 6.

#### 3.1.2. The Effect of Deformation Temperature

**Figure 7:**
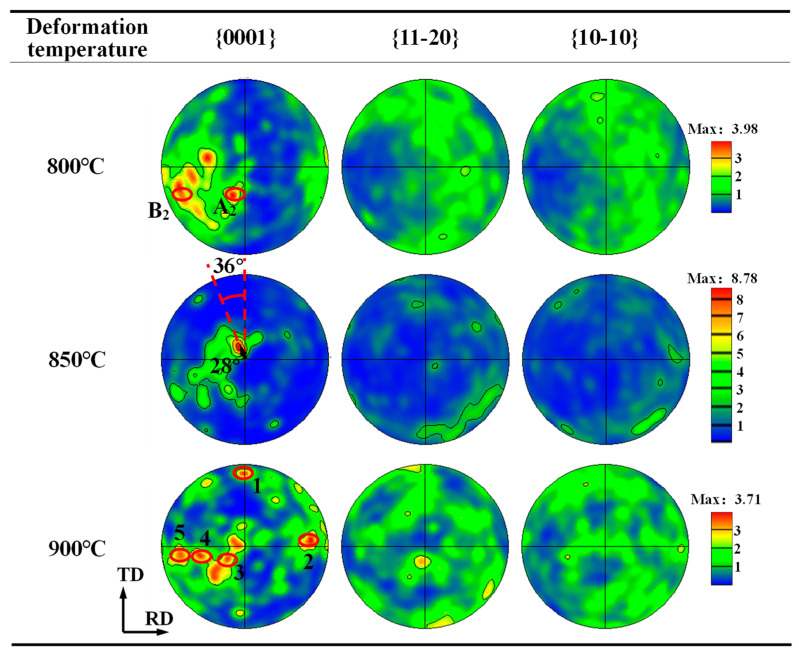
PF of α phase under different plane strain compression temperatures.

Figure 7 gives the PF of the α phase under various temperatures at a 60% deformation amount and 0.01 s^−1^ strain rate. As can be seen, when the temperature is 800 °C, each type of pole is dispersive, and some {0001} poles are distributed near the ND, as shown by the region A_2_ marked in Figure 7, while many {0001} poles are far away from the ND, as shown by the region B_2_ displayed in this figure. As the temperature rises to 850 °C, the orientation concentration of the α grain is enhanced. In this case, the angle between the C-axis of most grains and the ND can reach 28°, and the angle between the C-axis and the TD is around 36°. The {10-10} and {11-20} poles are located in the RD-TD plane (Figure 7), presenting a non-standard basal texture component. At a temperature of 900 °C, the pole becomes dispersed again, as illustrated in Figure 7. Here, the {0001} pole presents five clustered zones, and the {10-10} and {11-20} poles are almost uniformly distributed in the RD-TD plane, so the texture intensity is low.

The above orientation features are first attributed to the slip behavior under various deformation temperatures. As mentioned in Section 3.1.1, the basal slip is the most easily operated system of α-Ti during hot deformation. This is because the deformation resistance of the basal slip is lower than that of the pyramidal slip under a compression sate. When the temperature is low, although the basal slip is dominant, the critical shearing stress (CRSS) is high. Therefore, only the slip in the grain with a soft orientation can operate and promote grain orientation evolution. Conversely, the basal slip is difficult to initiate in the hard-oriented grain, and the grain orientation of this grain is close to its initial orientation. Therefore, the overall texture is rather dispersive. With the increase in temperature, the CRSS of the basal slip decreases, resulting in a large number of activated basal slips; thus, the orientation becomes concentrated. When the deformation temperature is high, i.e., 900 °C, the CRSS of the pyramidal <a> and <a + c> also decreases significantly [27,28,29]. Therefore, under the same load, the possibility of these slip systems being activated enhances. It has been reported that the pyramidal <a> slip can make the {10-10} poles to shift to the tension direction and the pyramidal <a + c> slip will make the C-axis of the grain rotate to the tensile direction [7,8]. This may be a reason for the dispersive and weak texture of the TA15 alloy after high-temperature plane strain deformation.

**Figure 8:**
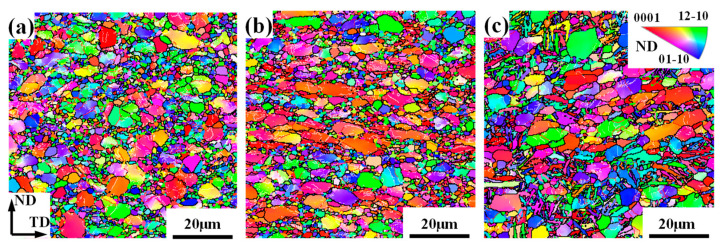
IPF maps of a TA15 alloy under different plane strain deformation temperatures: (**a**) 800 °C; (**b**) 850 °C; and (**c**) 900 °C.

Figure 8 gives the overall microstructure characteristics under different deformation temperatures. It can be seen that, at a temperature of 800 °C, even if many refined grains are generated, the morphology of α_p_ still keeps an equiaxed shape, which is close to the initial microstructure displayed in Figure 2. As the deformation temperature increases, the axial ratio of α_p_ obviously increases, and the refinement degree by DRX at 850 °C is higher than that at 900 °C. In addition, at a temperature of 900 °C, a lot of lamellar α (α_l_) appear and their volume fraction can reach 13%, which indicates that phase transformation is generated under this condition.

**Figure 9:**
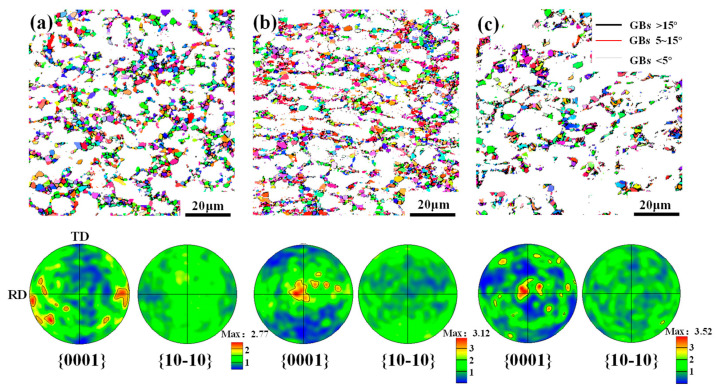
DRX grains and their {0001} and {10-10} PFs of a TA15 alloy under different plane strain deformation temperatures: (**a**) 800 °C; (**b**) 850 °C; and (**c**) 900 °C.

To explore the role of DRX on texture formation with temperature, IPF and PF maps of DRX grains were obtained, as shown in Figure 9. As can be seen, the DRX kinetics is similar at 800 °C and 850 °C, which is approximately 37%. Meanwhile, the size of DRX grains at 800 °C is greater than that at 850 °C. This is because DDRX is difficult to start at a lower temperature. Consequently, the dominant DRX mechanism is CDRX at 800 °C. In terms of CDRX grains, their size primarily depends on the substructure formed during hot deformation, so it is higher than that of DDRX grains. The similar texture characteristics of overall grains and DRX grains in Figure 7 and Figure 8a can also confirm the prevalence of CDRX at 800 °C. When the temperature rises to 850 °C, the activation energy of DRX decreases, which promotes the nucleation and growth of DDRX. So, the mean size of DRX grains decreases. Comparing Figure 7 and Figure 9b, it can be found that the orientation of DRX grains is similar to that of overall grains, which suggests that CDRX is still the dominant DRX mechanism, although DDRX is gradually obvious. When the temperature is 900 °C, the DRX fraction decreases to 22%. The clustered zones of DRX grains are always similar to those of the whole texture. These results indicated that CDRX is always the dominant DRX mechanism even if DDRX is gradually more common with the increase in temperature.

**Figure 10:**
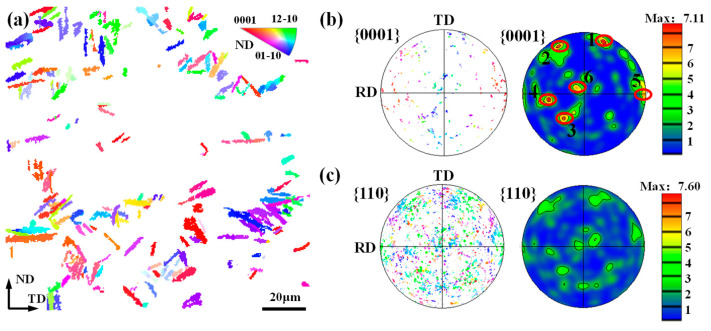
(**a**) IPF of α_l_, PF of (**b**) α_l_ and (**c**) β phase at a deformation temperature of 900 °C.

With regard to phase transition, it is well known that when the deformation temperature exceeds the α/β phase transition point, the α phase will be transformed into the β phase with six orientations according to the Burgers orientation relationship (BOR) [30]. Inversely, during the cooling process, the β phase will be transformed into an irregular elongated α phase with 12 kinds of orientations. The detailed orientation of the α grain is determined by the microstructure and processing conditions. In the present work, it is interesting to find that the phase transition occurs at a temperature of 900 °C, which is lower than the phase transition temperature. Such a phenomenon may be attributed to the following reasons. During plane strain compression, deformation heat will cause the temperature to rise. Therefore, at 900 °C, the temperature in the central region of the sample may exceed the beta-transition temperature, resulting in the α→β phase transformation. At the air-cooling stage, α_l_ is graduallu precipitated from the β phase, finally leading to the microstructure shown in Figure 8c. In order to investigate the effect mechanism of phase transition on texture evolution, the IPF of most α_l_ and the PF of α_l_ and the β phase at 900 °C were obtained, as shown in Figure 10. It can be seen that there are six obvious {0001} clustered zones and these zones corresponds to the {110} poles of the β phase (Figure 10c), which means six distinct α variants are produced in the process of β→α. Meanwhile, it is noted that the intensity of variants in regions 1 to 4 is much greater than that in regions 5 and 6. Compared with Figure 6c and Figure 10b, the orientation of the clustered area of α_l_ corresponds exactly to that of the whole texture, which implies that the phase transition is a key factor affecting the grain orientation evolution at 900 °C. Combining Figure 9c and Figure 10b, it is found that only the {0001} poles of α_l_ in region 5 present similar orientation to that of DRX grains. This is because when the orientation of the α_p_ and β phase meets the BOR, the C-axis of α_l_ generated by phase transformation is often same as that of α_p_ [31], whose orientation is close to the DRX grain orientation. Based on the orientation of overall grains, DRX grains and α_l_ at 900 °C, it can be concluded that the orientation of overall grain is similar to that of α_l_, so the influence of phase transformation on texture is much greater than that of DRX on texture, and multi-orientation of α_l_ produced by phase transition can effectively weaken the deformation texture.

#### 3.1.3. The Effect of Strain Rate

**Figure 11:**
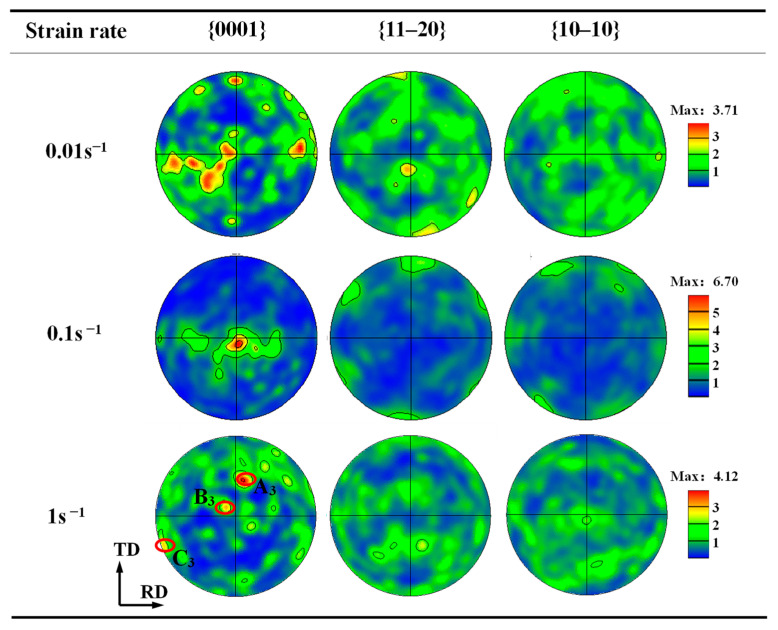
PF of α phase under different strain rates.

Figure 11 displays the PF of the α phase under various strain rates at a 60% deformation amount and a 900 °C deformation temperature. As we can see, with the strain rate increasing, the poles first gather and then disperse. The basal texture component at a strain rate of 0.1 s^−1^ is the most obvious and strongest. When the strain rate increases to 1 s^−1^, the {0001} poles have three distinct clustered regions, A_3_, B_3_ and C_3,_ as shown in Figure 11. Here, the C-axis of region B_3_ has an angle of 38° with the ND and an angle of 11° with the TD. This phenomenon may be because the operation of other slip systems increases with strain rate, except for the basal slip, to coordinate a faster plastic deformation during TMP.

**Figure 12:**
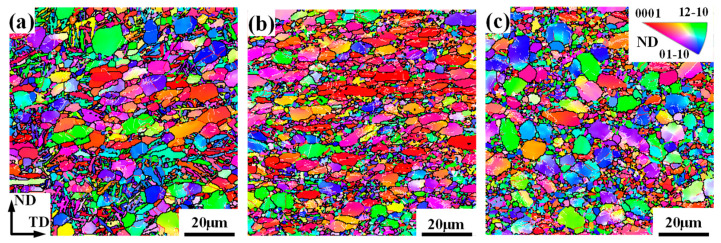
IPF maps of a TA15 alloy under different strain rates: (**a**) 0.01 s^−1^; (**b**) 0.1 s^−1^; and (**c**) 1 s^−1^.

**Figure 13:**
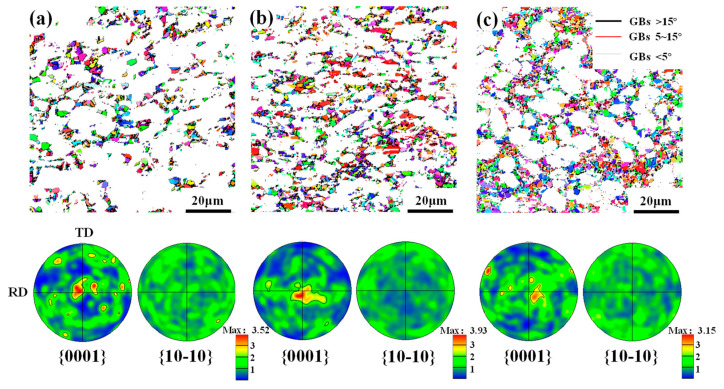
DRX grains and their {0001} and {10-10} PFs of a TA15 alloy under different strain rates: (**a**) 0.01 s^−1^; (**b**) 0.1 s^−1^; and (**c**) 1 s^−1^.

Figure 12 and Figure 13 give the microstructural evolution of overall grains and DRX grains at different strain rates, respectively. In Figure 12, α_l_ appears at a strain rate of 0.01 s^−1^, which is the reason for the dispersive texture feature of overall grains in this case. However, as the strain rate increases to 0.1 s^−1^, the microstructure after deformation mainly consists of coarsen α_p_ grains and refined tiny α grains, and α_p_ is evidently compressed along the ND. This result indicates that the high strain rate is not good for phase transformation. This is because a higher strain rate can significantly reduce the deformation time, so the α→β transformation has not started significantly yet while the temperature decreases rapidly due to air cooling. Thus, the phase transformation is not obvious. Correspondingly, there is little α_l_ observed under a strain rate of 0.1 s^−1^ and 1 s^−1^ at a temperature of 900 °C, as shown in Figure 12b,c. The orientation of DRX grains (Figure 13b) at a strain rate of 0.1 s^−1^ is similar to that of deformed grains, which implies that CDRX is prevalent under this condition. When the strain rate is 1 s^−1^, the α_p_ morphology does not change significantly compared to the initial microstructure, which may be caused by the following reason. A high strain rate easily induces significant inhomogeneous deformation, which makes the substructure at GBs preferentially rapidly rotate to coordinate the plastic deformation, further producing CDRX, while the substructure in the interior of coarse α_p_ is not fully deformed. Therefore, the orientation of coarse α_p_ is rather dispersive (Figure 12c), and the orientation of DRX grains produced along GBs is similar to that of deformed grains. That is to say, CDRX is still most common at a higher strain rate. Similarly, its effect on texture evolution is limited.

### 3.2. Mechanical Properties under Different Deformation Conditions

**Figure 14:**
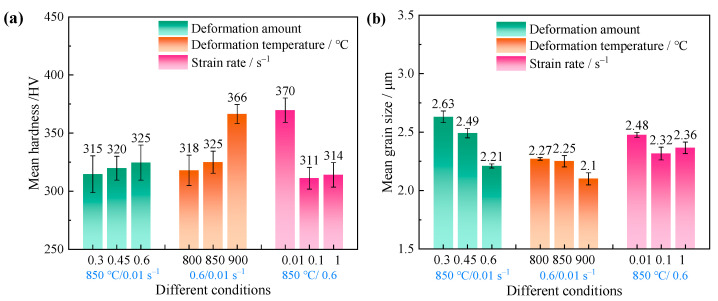
Distribution of (**a**) microhardness and (**b**) grain size under different forming parameters.

The microhardness distribution of a plane strain-compressed sample is displayed in Figure 14a. As we can see, microhardness can be gradually improved with an increase in the deformation amount and temperature. The average hardness is enhanced by 10 HV as the deformation increases from 30% to 60%, while it is improved by 40 HV as the temperature rises from 800 °C to 900 °C, which means that the deformation temperature has a more significant impact on the mechanical properties than the deformation amount. In terms of the increase in strain rate, the microhardness decreases first and then increases slightly.

**Figure 15:**
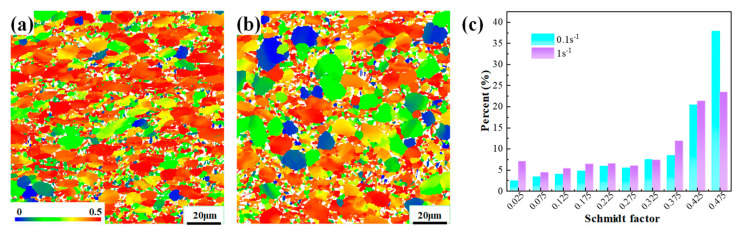
Schmidt factor maps of prism slip when loading along the RD at a strain rate of (**a**) 0.1 s^−1^ and (**b**) 1 s^−1^; (**c**) detailed distribution values of Schmidt factor.

For α titanium alloys, the mechanical performance after deformation is strongly depend on the grain size as well as the texture features. Figure 14b gives the average grain size after plane strain compression through EBSD data. It is found that the average grain size decreases with the increase in deformation amount and temperature; what is more, the refinement degree under different deformation amounts is better than that under different temperatures. That is to say, grain size is more sensitive to deformation amount, whereas the microhardness strongly relies on the deformation temperature. By comparing the microstructures in Figure 5 and Figure 8, it can be found that the microhardness is relatively low at the equiaxed α morphology. If α_l_ are produced, the microhardness is significantly improved. This is because a higher GB content in a lamellar microstructure can effectively improve the material strength [32]. In addition, with the increase in strain rate, the average grain size decreases first and then increase, which shows a similar tendency to the change in microhardness. In other words, the mechanical property evolution in this case does not meet the Hall–Petch relationship. The reason may be as follows. From a 0.01 s^−1^ to 0.1 s^−1^ strain rate, the decrease in microhardness is mainly caused by the change in α grain morphology. As the strain rate increases from 0.1 s^−1^ to 1 s^−1^, the deformation texture may play a more important role in microhardness than grain size. As shown in Figure 11, an obvious basal texture is generated at a strain rate of 0.1 s^−1^, while the grain orientation is rather dispersive at a strain rate of 1 s^−1^. When the material loads along the RD at room temperature, the prism slip is the dominant deformation mechanism [6,33,34]. In this case, the Schmidt factor of the prism slip at 0.1 s^−1^ is greater than that at 1 s^−1^, as shown in Figure 15, which means the prism slip is easily activated at a strain rate of 0.1 s^−1^, thus exhibiting a lower microhardness.

## 4. Conclusions

In this study, the texture formation mechanism of a TA15 alloy during hot plane strain compression was investigated under different forming conditions by analyzing the slipping behavior and microstructural evolution phenomena. Further, the microhardness caused by the microstructure and texture were explored. The detailed conclusions are drawn as follows:

(1) After plane strain compression, the basal texture component appears regardless of different deformation amounts, which is because the massive basal slip activated makes the C-axis of the α grain rotate to the compression axis. With the increase in deformation, the {0001} poles of the α grains first approach the ND and then deviates from the ND. The deviation is due to the inhomogeneous basal slip at a small deformation amount as well as the higher principal strain ratio at a large deformation amount. Meanwhile, both DDRX and CDRX grains are observed, and the dominant DRX mechanism changes from DDRX to CDRX with deformation. However, DRX has little effect on the texture evolution due to a lower fraction of DDRX grains and a similar orientation of CDRX grains to the deformed grain.
(2) With the increase in deformation temperature, the basal texture component first strengthens and then weakens. At a deformation temperature of 800 °C, the dispersive orientation of the α grains may be associated with a smaller amount of activated basal slip since the critical shear stress (CRSS) of this slip is relatively high under this condition. At the temperature of 850 °C, the decrease in CRSS promotes basal slip, so the intensity of the basal texture becomes strong. However, as the deformation increases to 900 °C, the deformation heat effect in this condition may make the temperature in the central region of the sample higher than the beta-transition temperature, so α→β phase transformation occurs during deformation and lamellar α (α_l_) precipitate during the cooling stage. The occurrence of lamellar α disperses the overall grain orientation; thus, the basal texture feature is obviously weakened. In addition, CDRX also prevails under different temperatures while it has little effect on the texture evolution.
(3) With the increase in strain rate, the orientation of the α grain first comes together and then disperses. At a lower strain rate, the existence of α_l_ is the main reason for the dispersive texture. At a higher strain rate, the operation of other slip systems will increase, beside basal slip, to coordinate faster plastic deformation. Moreover, significant inhomogeneous deformation makes the orientation of the substructure at GBs preferentially rotate, while the substructure in the interior of coarse α_p_ is not fully deformed. Therefore, there is little change in the α_p_ morphology and orientation of coarse α_p_ is rather dispersive. Similarly, CDRX is still most common at different strain rates but its influence on the texture evolution is limited.
(4) Microstructure type is the most critical factor to determine the microhardness of samples after plane strain compression deformation, and the formation of α_l_ can significantly improve the microhardness. As the α grain is mainly equiaxed-type, there is an obvious basal texture component after deformation, and the microhardness is inversely proportional to the grain size. However, as the orientation of equiaxed α is dispersive, the microhardness will be higher due to the lower Schmidt factors of the prism slip activated at room temperature.

## Figures and Tables

**Table 1:** The scheme of plane strain compression tests.

No.	Deformation Temperature (°C)	Strain Rate (s^−1^)	Deformation Amount (%)
1	800	0.01	60
2	850	0.01	30
3	850	0.01	45
4	850	0.01	60
5	900	0.01	60
6	900	0.1	60
7	900	1	60

## Data Availability

The original contributions presented in the study are included in the article, further inquiries can be directed to the corresponding author.
